# Flexible Humidity Sensors Based on Multidimensional Titanium Dioxide/Cellulose Nanocrystals Composite Film

**DOI:** 10.3390/nano12121970

**Published:** 2022-06-08

**Authors:** Xin Tong, Hong Wang, Huiyang Ding, Jing Li, Huifang Zhao, Zhaoyun Lin, Hongxia Xi, Xuejin Zhang

**Affiliations:** 1 Key Laboratory of Recycling and Eco-Treatment of Waste Biomass of Zhejiang Province, Zhejiang University of Science and Technology, Hangzhou 310023, China; xintong@zust.edu.cn (X.T.); 18855177898@163.com (H.W.); dinghuiyang0126@163.com (H.D.); ljing1987@gmail.com (J.L.); zhf9966@163.com (H.Z.); 2 Key Laboratory of Pulp and Paper Science & Technology of Ministry of Education, Qilu University of Technology (Shandong Academy of Sciences), Jinan 250353, China; linzhaoyun1233@126.com; 3 School of Chemistry and Chemical Engineering, South China University of Technology, Guangzhou 510640, China; cehxxi@scut.edu.cn

**Keywords:** humidity sensor, TiNP/CNC-based humidity sensors, flexible, density functional theory

## Abstract

A humidity sensor is a crucial device in daily life; therefore, in the present study, a novel humidity sensor was designed to increase its specific surface area to improve its humid sensing capacity and conductivity. Titanium dioxide nanoparticles (TiNP) consisting of zero-dimensional nanospheres and one-dimensional nanotubes were prepared by anodic oxidation. Rod-shaped cellulose nanocrystals (CNCs) with average length and diameter of 60 nm and 800 nm, respectively, were obtained by enzymatic hydrolysis and high pressure homogenization. TiNP/CNC composite films exhibited superior hydrophilicity and large specific surface areas based on Fourier transform infrared spectroscopy and nitrogen adsorption–desorption results. The humidity sensing characteristics of sensors based on TiNP/CNC flexible composite films with varying contents of TiNP were investigated under a relative humidity range of 11–97%. The 6% TiNP/CNC-based humidity sensor exhibited high humidity response, rapid response/recovery speed, and high stability. Furthermore, the humidity sensing mechanism of TiNP/CNC composite films was analyzed based on the density functional theory. TiNP/CNC-based humidity sensors could be applied in flexible and wearable electronics.

## 1. Introduction

Humidity (water vapor) is one of the key meteorological elements that indicate the amount of water vapor in the air and the degree of wetness. Humidity regulation influences many aspects of daily life, such as human health, living environment, and product storage [1,2,3]. Therefore, humidity measurement is widely used in several sectors related to our daily life. Relative humidity (RH) is the ratio of the current water vapor pressure to the total pressure of saturated vapor, which can indicate water vapor saturation [4]. For resistive humidity sensors, resistance varies with an increase in RH, where humidity-sensitive materials are susceptible to adsorption and desorption of water molecules from the atmosphere. Although the most common construction for resistive humidity sensors includes a ceramic substrate coated with sensitive materials, metal, and heater electrodes [5], different types of humidity sensors have been developed based on flexible substrates [6], inkjet-printed textile, and textile fiber composite materials [7]. With the recent development of smart wearable devices, flexible humidity sensors have received increasing attention.

In general, in addition to humidity sensors possessing excellent humidity sensing properties, flexible humidity sensors fabricated with humidity-sensitive materials, interdigital electrode parameters, and flexible substrates, have a unique role in promoting the development of wearable devices [8]. To achieve excellent flexibility, multifarious ductile materials, including polyethylene terephthalate [1], polyimide [9], polyethylene naphthalate [10], and polydimethylsiloxane [11] have been utilized as substrates for wearable electronic devices. However, polymer film-based sensors have limited moisture adsorption capacity and breathability, which considerably reduce their sensitivity and comfort. An effective strategy for addressing the challenge is using biomass-derived cellulose as a sensor substrate.

Some researchers have observed that cellulose generally occurs in the form of cellulose fiber, cellulosic paper, or cellulosic film, which when combined with other materials creates a conductive network for humidity sensors [12]. However, cellulose-based sensors are associated with certain disadvantages, such as low mechanical strength, lack of repeatability and stability, among others. Cellulose nanocrystal (CNC), a material derived from cellulose fibers, is a potential scaffold material because of the numerous hydroxyl groups that exist in its molecular chains. CNCs can interact with water molecules through hydrogen bonding. In addition, CNCs have hydrophilic surfaces but do not swell in water [13]. Furthermore, the remarkable properties of CNCs, including high mechanical strength with Young’s modulus of approximately 150 GPa and tensile strength of 10 GPa, large surface area, abundant sources, renewability, biodegradability, biocompatibility, and non-toxicity make the CNCs suitable for use as a base for humidity sensors [14].

Although CNCs can act as effective scaffold materials for humidity sensing, the choice of humidity-sensitive materials also directly affects sensor performance. To date, a variety of materials, including porous ceramics, semiconducting metal oxides, polymers, metallic nanowires, and carbon-based nanomaterials, have been explored as humidity sensing materials [6,15]. Titanium dioxide (TiO_2_) is one of the well-known semiconductor oxides, and has attracted the attention of researchers due to its remarkable properties, including low cost, non-toxicity, and high stability [16]. Furthermore, TiO_2_ can be a suitable candidate for humidity sensing due to its high responsive conductivity and hydrophilic properties. Notably, the sensing properties of TiO_2_ materials largely depend on their morphologies, which in turn, are determined by the preparation methods [17]. The geometry of TiO_2_ nanomaterials can vary from quantum dots, nanofibers, nanotubes, nanosheets to nanoflowers, and other nanostructures depending on their various synthesis methods, such as hydrothermal synthesis, template-assisted synthesis, and chemical decomposition, among others [18]. TiO_2_ nanomaterials with relatively large surface areas have numerous porous sites and exhibit better electron transitions, which implies that they may have superior humidity sensing properties.

In the present study, multidimensional TiO_2_ nanoparticles (TiNP) consisting of zero-dimensional (0-D) nanospheres and one-dimensional (1-D) nanotubes were synthesized by anodic oxidation. They can provide several adsorption channels for H_2_O molecules and facilitate the rapid adsorption and desorption of H_2_O molecules. The CNCs were obtained by enzymatic hydrolysis combined with high pressure homogenization. The biodegradable CNC materials were used for the preparation of an integrated humidity sensor to enhance the mechanical properties of the composite material and to further improve the sensing properties of the sensor because of the water molecules adsorption capacity of CNCs. Afterwards, material characterizations of TiNP/CNC composite films were performed using a field emission scanning electron microscope (FESEM), X-ray diffraction (XRD), Fourier transform infrared (FTIR) spectroscopy, and nitrogen (N_2_) adsorption–desorption analyses. Furthermore, humidity sensing properties of flexible humidity sensors fabricated using TiNP/CNC composite films at room temperature and RH of 11–97% were investigated. Finally, the humidity sensing mechanism of TiNP/CNC composite films was analyzed based on the density functional theory (DFT).

## 2. Materials and Methods

### 2.1. Synthesis of Cellulose Nanocrystals and Titanium Dioxide Nanoparticles

Highly ordered and uniform TiO_2_ nanotube arrays were initially grown via electrochemical anodization of Ti metal foil (0.1 mm) grown in a glycol solution with 0.55 wt% ammonium fluoride and 20 wt% deionized water at 30 V for 6 h. Subsequently, the anodized Ti foil was annealed at 450 °C for 2 h. The calcined nanotube arrays were then broken down and ground manually to obtain TiNP with residual nanotube fragments.

After swelling in 50% aqueous propanetriol solution for 2 h, the cotton pulp was pretreated by enzymatic hydrolysis. Based on a total enzyme concentration of 10 U/mL, with cellulase and xylanase combined at a concentration ratio of 9:1, the pretreated pulp was obtained following hydrolysis at 50 °C for 12 h, followed by successive washing and centrifugation cycles. Afterward, final CNC suspensions were sonicated in a high pressure micro jet homogenizer (LM20, Microfluidics International Corporation, Westwood, MA, USA) at 25,000 psi for 15 min.

### 2.2. Fabrication of Humidity Sensors

The aqueous CNC obtained was then diluted with ultrapure water to a concentration of 0.1 wt%. Thereafter, CNC dispersions (containing 0.1 wt% CNCs, 50 mL) were blended with TiNP and dispersed using an ultrasonic disruptor (JY99-IIDN, Ningbo Scientz Biotechnology Co., Ltd., Ningbo, China) at 50% amplitude for 5 min.

**Figure 1:**
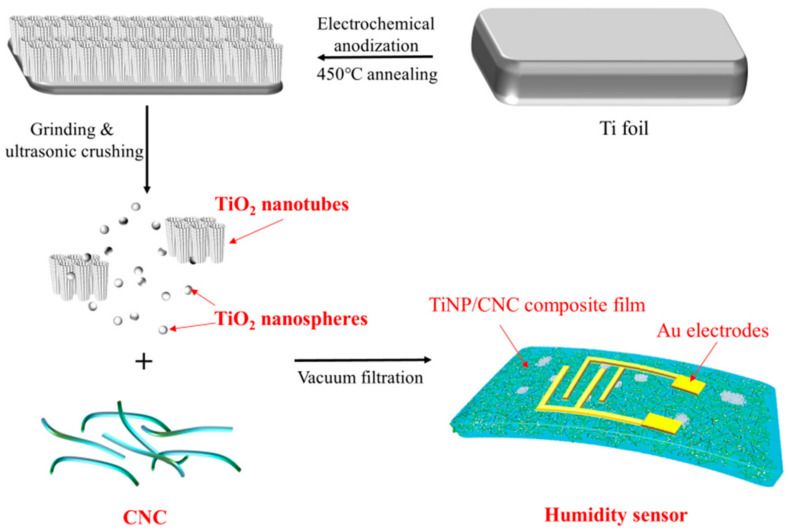
A schematic diagram of the TiNP/CNC-based humidity sensor preparation process.

Subsequently, the TiNP/CNC composite films with various ratios were prepared by vacuum-assisted filtration using polyvinylidene fluoride membrane filters with pore sizes of 0.22 μm. After filtration, the formed TiNP/CNC gels were pressed to dry in an ambient laboratory atmosphere and then peeled off from the filter membranes. The TiNP/CNC sensors were developed by processing gold interdigitated electrodes on the composite films based on ion sputtering. The synthesis and fabrication processes of TiNP/CNC-based humidity sensors are illustrated in Figure 1.

### 2.3. Material Characterization

The surface morphology of the TiNP/CNC nanocomposite films was analyzed using a field emission scanning electron microscope (Zeiss GeminiSEM 500, Oberkochen, Germany), which was equipped with an energy-dispersive spectrometer for characterizing the chemical composition of TiNP/CNC nanocomposite films. XRD patterns were measured using an XRD spectrometer (D8 Advance, Bruker AXS, Karlsruhe, Germany) with Cu Kα radiation operated at 40 kV and 40 mA in the 2θ range of 10°–80°.

FTIR spectroscopy was used to identify functional groups in the TiNP/CNC nanocomposite films in the 4000–800 cm^−1^ region, and was performed using a FTIR spectrometer (VERTEX 70, Bruker Optics GmbH, Ettlingen, Germany). The N_2_ adsorption–desorption experiments were performed using an automatic gas adsorption analyzer (ASAP 2020 Plus HD 88, Micromeritics Instrument Corp, Norcross, GA, USA), with samples being degassed at 60 °C for 12 h to determine TiNP/CNC nanocomposite film porosity and specific surface area. Specific surface area was calculated using the Brunauer–Emmett–Teller (BET) equation and pore size distribution was calculated based on the Barrett–Joyner–Halenda (BJH) method.

### 2.4. Humidity Sensing Test

The characteristics of TiNP/CNC-based humidity sensors under various humidity conditions were determined by placing them in series of closed bottles with saturated solutions of different salts at room temperature. The various humidity conditions created by saturated solutions of different salts were as follows: LiCl (11%), MgCl_2_ (33%), KCO_3_ (43%), MgNO_3_ (54%), KI (67%), NaCl (75%), KCl (84%), and KNO_3_ (97%). To achieve optimized sensing performance, six groups of TiNP concentrations (0, 1, 3, 6, 9, and 12 wt%) were used to fabricate the sensors, and the electrical signals were measured using a gas sensing measurement system (WS-30A, Weisen Electronics Technology Co., Ltd., China).

The response of the TiNP/CNC-based humidity sensor was calculated using Equation (1) as follows:

S = R_0_/R (1)

where R_0_ is the initial resistance of the sensor at 11% RH and R is the final resistance at testing RH level.

### 2.5. Calculation Method

To explore the adsorption behavior of water (H_2_O) molecules on TiO_2_ surface, a representative (101) surface of anatase TiO_2_ thin film was considered [19,20,21]. Therefore, in our calculations, anatase TiO_2_ (101) surface was modeled by a periodic (2 × 2) slab with two O–Ti–O layers.

The anatase TiO_2_ (101) surface has two types of atoms: the topmost atoms are five-fold coordinated Ti atoms (Ti_5c_, hereafter referred to as Ti^4+^) and two-fold coordinated O atoms (O_2c_), which have the highest probability of reacting with gases [22]. The other atoms considered in the bulk position were six-fold coordinated Ti atoms (Ti_6c_) and three-fold coordinated O atoms (O_3c_).

All first principle calculations were performed using the Cambridge Sequential Total Energy Package in Materials Studio 8.0. Generalized Gradient Approximations was used to determine the exchange–correlation energy. The valence electron grouping selected for each element of Ti, O, and H were Ti 3s^2^3p^6^3d^2^4s^2^, O 2s^2^2p^4^, and H 1s, respectively. To calculate the adsorption energy, E_ads_ of H_2_O molecules on anatase TiO_2_ (101) surface, the following expression was used:

E_ads_ = E_(slab+H_2_O)_ − [E_(slab)_ + E_(H_2_O)_] (2)

where E_(slab+H_2_O)_ is the total energy of H_2_O molecules adsorbed on anatase TiO_2_ (101) surface, while E_(slab)_ and E_(H_2_O)_ are the isolated surface and H_2_O molecule energies, respectively.

## 3. Results and Discussion

### 3.1. Morphological and Structural Characteristics

**Figure 2:**
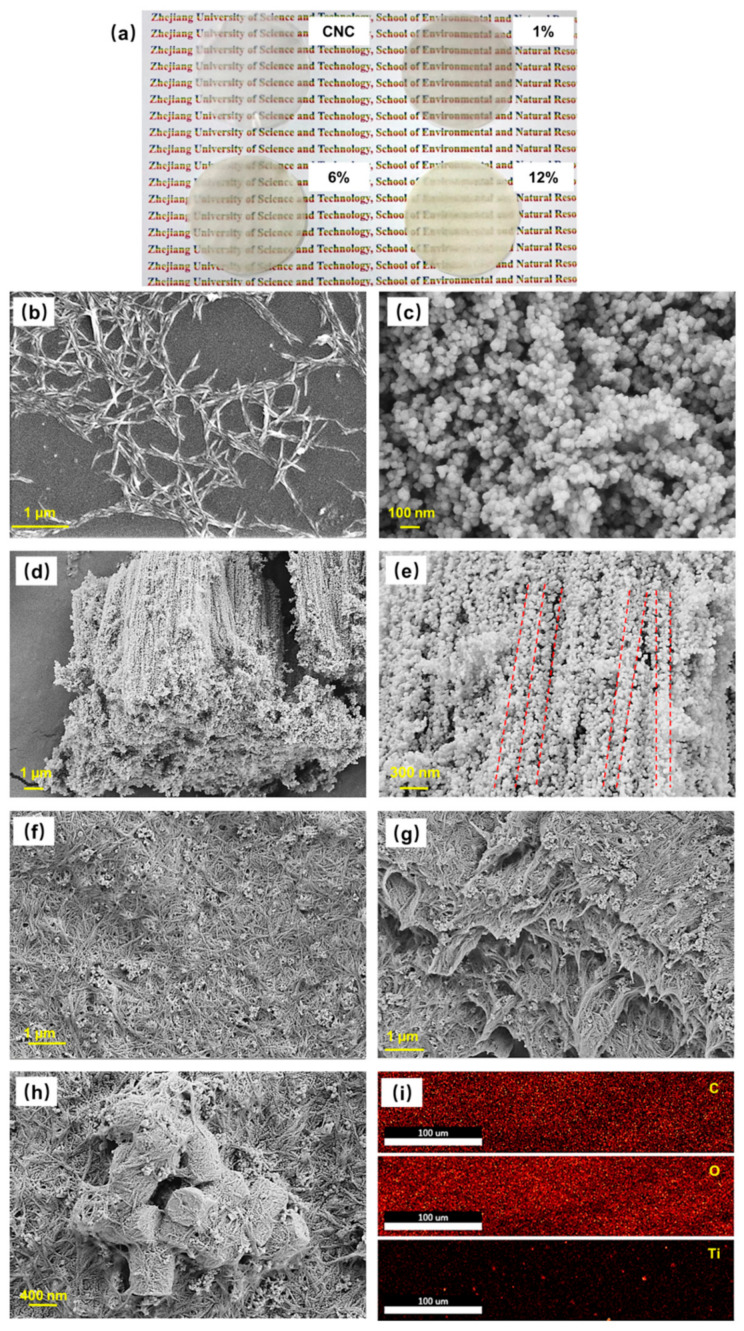
(**a**) Pictures of TiNP/CNC composite films with different TiNP content. Scanning electron microscopy (SEM) images of (**b**) cellulose nanocrystals (CNC), (**c**) spherical titanium dioxide (TiO_2_) nanoparticles (TiNP), (**d**,**e**) TiO_2_ nanotubes, (**f**,**g**) top view, side view of 6% TiNP/CNC composite film, (**h**) 12% TiNP/CNC composite film. (**i**) SEM elemental mapping images of C, O, and Ti elements of 6% TiNP/CNC composite film.

Morphology and structure are directly associated with humidity sensing properties of composite materials. Pictures and SEM images of CNCs and TiNP/CNC composite films are illustrated in Figure 2.

As shown in Figure 2a, the CNC film is transparent and clearly shows the text below it. As the content of TiNP increases, the transparency of the composite films decreases. Rod-shaped CNCs with average length and diameter of 60 nm and 800 nm, respectively, are obtained in the present study (Figure 2b). A further examination of the TiNP structure revealed two different TiO_2_ nanoparticle structures. During the anodic oxidation process, the nanotube arrays are ground manually and broken down via ultrasonic treatment to form 0-D spherical nanoparticles with an average diameter of 60 nm (Figure 2c). Moreover, some of the TiO_2_ nanotubes retain their original structure, in turn, forming 1-D honeycomb porous TiO_2_ arrays with an average pore diameter of 200 nm, as illustrated in Figure 2d. Notably, the tube walls of individual TiO_2_ nanotubes are composed of TiO_2_ nanospheres (Figure 2e), which can be attributed to two types of bonding forces involved in the formation of bulk nanotube arrays. One of the bonding forces is that existing between TiO_2_ crystals formed on nanotube walls and the other exists between the walls of the nanotubes [23]. In addition, the different structures of TiO_2_ are fabricated with CNCs to form composite films. TiNPs are uniformly distributed among the CNCs, forming composite films with flat surfaces (Figure 2f). The film is observed as a multilayered structure from the side-view of the composite film (Figure 2g). However, when the content of TiNP is increased to 12%, the tube clusters will stack on the film surface (Figure 2h). A combination of elemental mapping images in Figure 2i with the SEM images reveals that the spherical TiO_2_ nanoparticles are completely interwoven among the CNCs while the bulky TiO_2_ nanotube arrays are uniformly distributed in the composite film. The special composite structure of TiNP/CNC nanocomposite film can provide a large surface area for the adsorption of H_2_O molecules, thereby allowing adsoprtion of more H_2_O molecules on the composite films and enhancing the sensing properties of humidity sensors.

**Figure 3:**
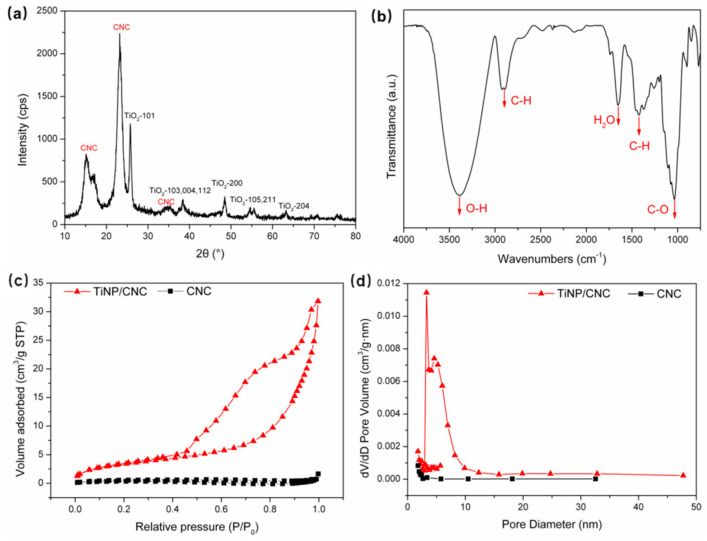
(**a**) X-ray diffraction pattern, (**b**) Fourier transform infrared spectroscopy spectrum, (**c**) nitrogen adsorption–desorption isotherms and (**d**) pore size distribution of 6% TiNP/CNC composite film.

XRD was used to test the crystal structure of the 6% TiNP/CNC composite films. XRD spectra of TiNP/CNC composite films are illustrated in Figure 3a. The sample exhibits characteristic diffraction peaks of TiO_2_ at 2θ = 25.4°, 37.1°, 37.9°, 38.6°, 48.1°, 54.0°, 55.1°, and 62.7°, which correspond to (101), (103), (004), (112), (200), (105), (211), and (204) crystal planes of anatase phase of TiO_2_, respectively (JCPDS-21-1272) [24]. Moreover, CNC exhibits peaks at 16.2°, 22.7° and 34.9°, which correspond to (101), (002), and (040) crystal planes of cellulose crystals [25,26]. Therefore, the results indicate that the crystalline structures of TiO_2_ and CNCs were not altered after forming a composite.

The FTIR spectrum of 6% TiNP/CNC composite film is illustrated in Figure 3b. The broad peaks observed between 3000 and 3500 cm^−1^ are ascribed to the -OH group absorption peaks, whereas those observed at 2894 cm^−1^ resulted from C–H stretching in methylene groups [26]. The characteristic absorption peaks of C–O in cellulose are observed at 1050 cm^−1^. The broad absorption band observed in the 3100–3600 cm^−1^ region can be attributed to the stretching mode of the -OH group. The -OH group is hydrophilic, and can indicate the capacity of a sample to adsorb H_2_O molecules [27].

To further verify the specific surface areas of the composite films, N_2_ adsorption–desorption experiments were performed. The N_2_ adsorption–desorption isotherms and pore size distributions of pure CNC and of 6% TiO_2_/CNC composite films are illustrated in Figure 3c,d, respectively. Based on the BET surface area analysis measurements, the surface areas of pure CNC and 6% TiNP/CNC composite films were 0.99 m^2^/g and 12.39 m^2^/g, respectively. The values indicated that the CNC films had considerably low porosity due to CNC aggregation. In contrast, 6% TiNP/CNC composite film exhibited relatively high surface areas, suggesting that TiO_2_ nanocrystals considerably hindered the stacking of CNC particles. The porous structure of TiO_2_ nanotubes also increases the specific surface area of the composite films. The 6% TiNP/CNC composite film exhibited type IV nitrogen adsorption isotherms when compared to pure CNCs (Figure 3c), suggesting the presence of a mesoporous structure [28]. Pore size distribution of pure CNCs and 6% TiNP/CNC composite films based on the BJH method are illustrated in Figure 3d. The average pore size of 6% TiNP/CNC composite film increased from 2.97 nm to 6.69 nm after forming a composite with TiO_2_. The unique structure of TiNP/CNC composite films can provide a relatively large surface area for the adsorption of more H_2_O molecules on TiNP/CNC composite film surfaces, which in turn, improves the response of the humidity sensor.

Based on the results of the present study, it can be concluded that TiNP/CNC composite films exhibit superior hydrophilicity and have high specific surface areas, which could enhance their humidity sensing capacities, especially low RH detection capacity.

### 3.2. Humidity Sensing Characteristics

The requirements for effective humidity sensors that measure variations in impedance after exposure to humidity in view of practical applications include the following: excellent sensitivity (i.e., the capacity to discriminate small variations in analyte concentrations over a wide range of RH values), short response and recovery times, and high repeatability [4]. Therefore, we analyzed the aforementioned sensor parameters and the results are discussed in the subsequent section.

The humidity sensing capacity of TiNP/CNC composite films was investigated based on a RH range of 11–97%, which was achieved by using various saturated salt solutions at room temperature.

**Figure 4:**
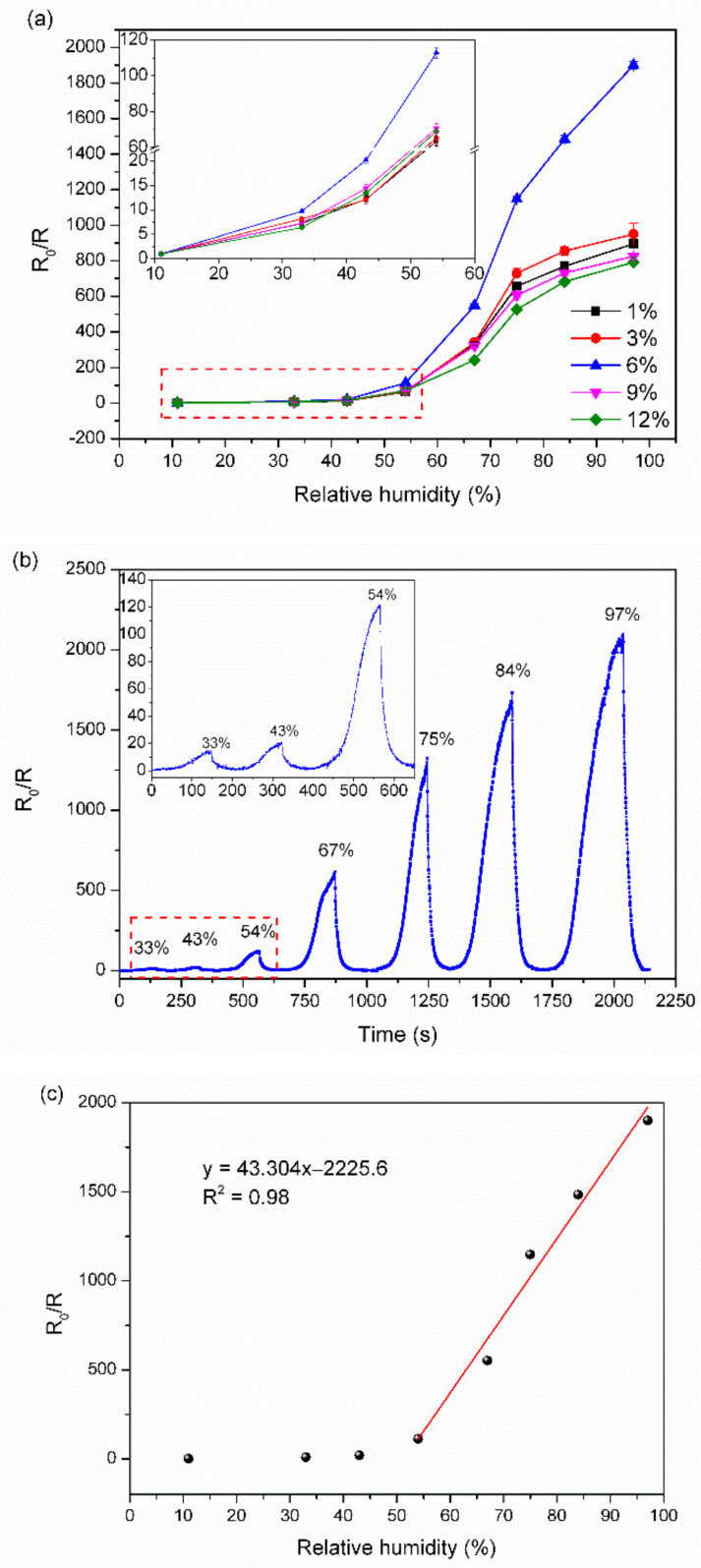
(**a**) Variations in humidity responses of TiNP/CNC composite film sensors with different TiNP contents. (**b**,**c**) Dynamic response and calibration curves of the 6% TiNP/CNC-based humidity sensors under 11–97% RH conditions.

Figure 4a shows humidity responses for TiNP/CNC composite film sensors with various TiNP contents (1–12 wt%). Humidity response values of all sensors increased with an increase in RH. The 6% TiNP/CNC-based humidity sensor had the capacity to detect small variations under low RH over a wide range of RH values. For sensors with varying TiNP contents (1–6%), higher responses were obtained with an increase in TiNP content of the composite film. However, as the TiNP content increased to 9%, humidity response decreased. The unique TiNP structure provides numerous gas contact channels to the composite membrane. In addition, as shown in Figure 2h, excess TiNP interwoven between CNCs blocks the gas contact channels and reduces the chances of H_2_O molecules coming into contact with the composite film structure; in turn, response of the sensors decreases considerably. The highest TiNP/CNC-based humidity sensor response was observed when TiNP content was 6%.

The dynamic response curve of the 6% TiNP/CNC-based humidity sensor under 11–97% RH conditions is illustrated in Figure 4b. The substantially high response of the 6% TiNP/CNC-based humidity sensor observed following exposure to less than 50% RH suggests that the sensor exhibits a rapid increase in response when exposed to high humidity. Figure 4c indicated that the 6% TiNP/CNC-based humidity sensor exhibited good linearity under the RH changing from 54% to 97% RH with the RH sensitivity at 41.57/% RH, which shows that the sensor is highly sensitive to the relative humidity.

**Figure 5:**
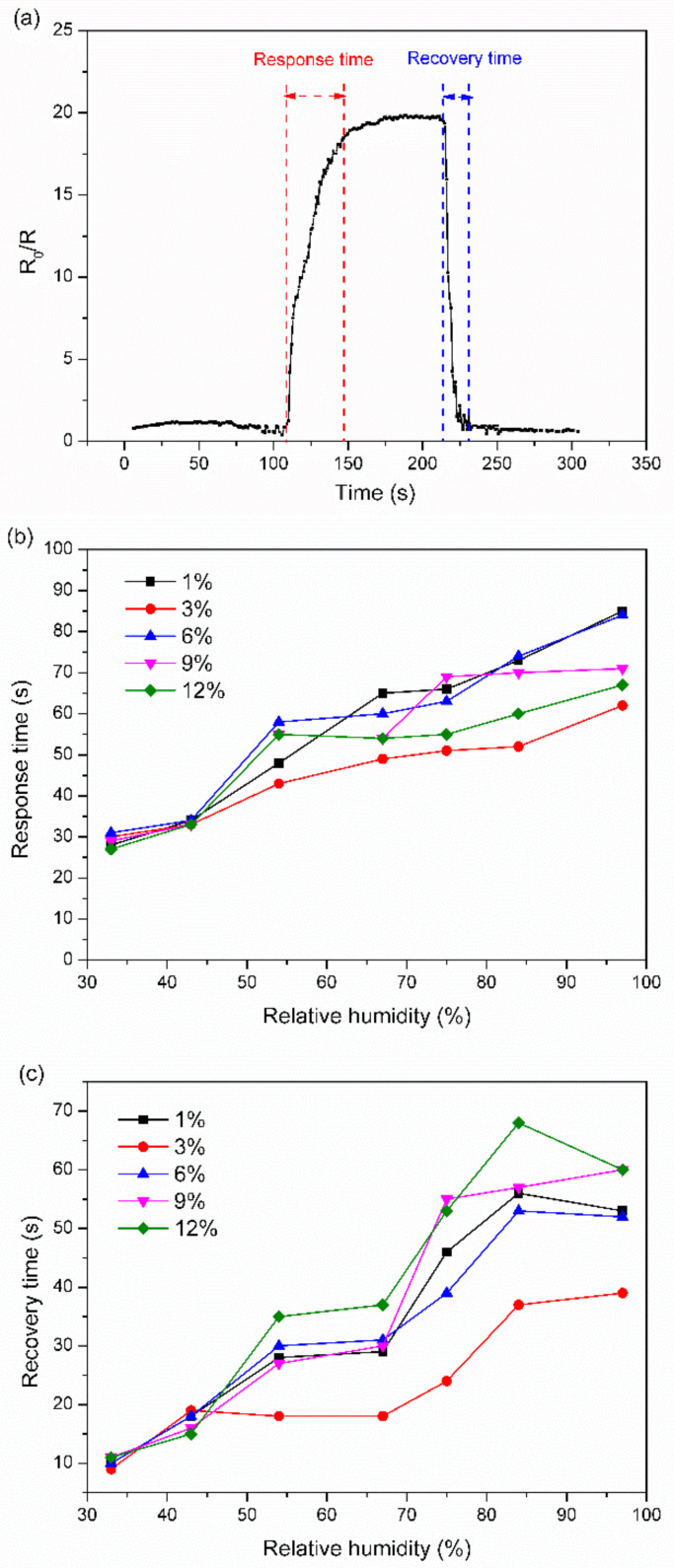
(**a**) Response-time curve of the 6% TiNP/CNC-based humidity sensors to 43% RH, (**b**) response time, and (**c**) recovery time of TiNP/CNC-based humidity sensors following exposure to 33–97% RH.

Figure 5a shows a single cycle of the response-time curve of the 6% TiNP/CNC-based humidity sensor to 43% RH. Response and recovery times are defined as the durations required by a sensor to achieve 90% of the total change in response during adsorption and desorption of H_2_O molecules, which are one of the key indicators of the practical application value of a sensor.

The response and recovery times for the 6% TiNP/CNC-based humidity sensors to 43% RH are 34 s and 18 s, respectively, as illustrated in Figure 5a. Figure 5b,c illustrates the response and recovery times of TiNP/CNC-based humidity sensors with various TiNP contents to 33–97% RH. The response and recovery times increased gradually with an increase in RH. Overall, recovery times were shorter than the response times, and the recovery times were within 20 s at less than 50% RH, suggesting that the TiNP/CNC-based humidity sensors have good reversibility.

Table 1 summarizes the performance of different flexible humidity sensors based on TiO_2_. It can be seen in Table 1 that the 6% TiNP/CNC-based humidity sensor has a high response value while maintaining short response and recovery times. In the TiNP/CNC composite films, CNCs can interact with H_2_O molecules through hydrogen bonding; the interwoven network of CNC and multidimensional structure of TiNP gives more channels for H_2_O molecules.

**Figure 6:**
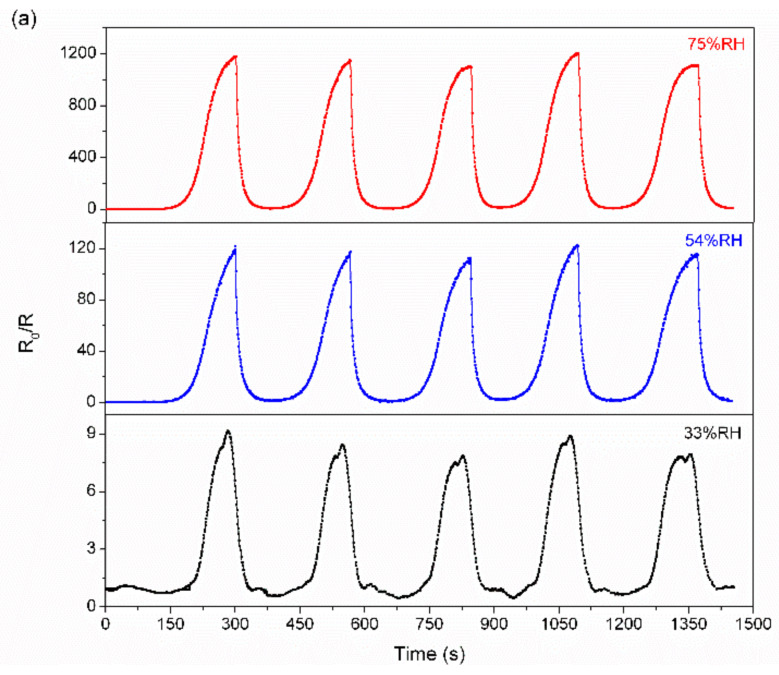

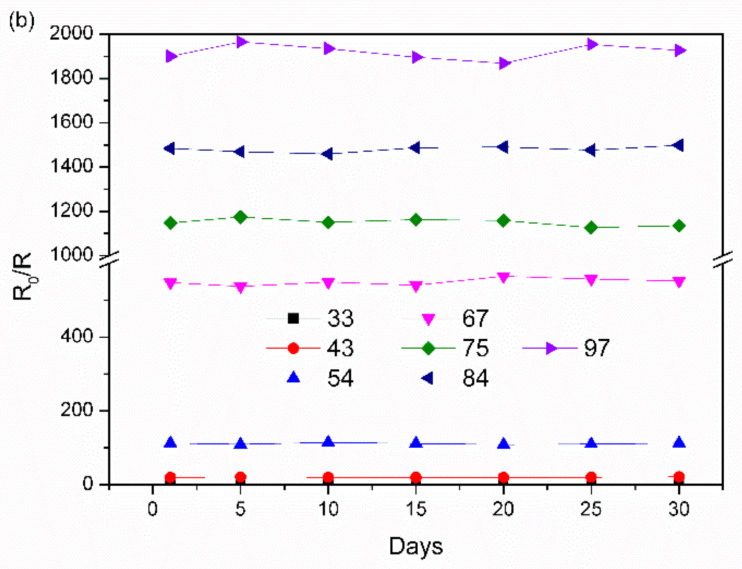
(**a**) Dynamic response curves of 6% TiNP/CNC-based humidity sensors to various relative humidity levels and (**b**) variations in response of 6% TiNP/CNC-based humidity sensors following exposure to 33–97% RH.

Repeatability and long-term stability are also key parameters of humidity sensors for practical sensing applications. The responses of the 6% TiNP/CNC-based humidity sensors are evaluated in five sensing cycles at 33%, 54%, and 75% RH (Figure 6a). The response curves of the sensors to the same RH values in different cycles are highly similar, which indicates good repeatability. Furthermore, the responses of 6% TiNP/CNC-based humidity sensor following exposure to 33–97% RH maintained for 30 days are illustrated in Figure 6b. A low standard deviation in response of the sensors under a definite humidity level indicates long-term stability.

### 3.3. Density Functional Theory Analysis and Sensing Mechanism of TiO_2_/CNC-Based Humidity Sensors

**Figure 7:**
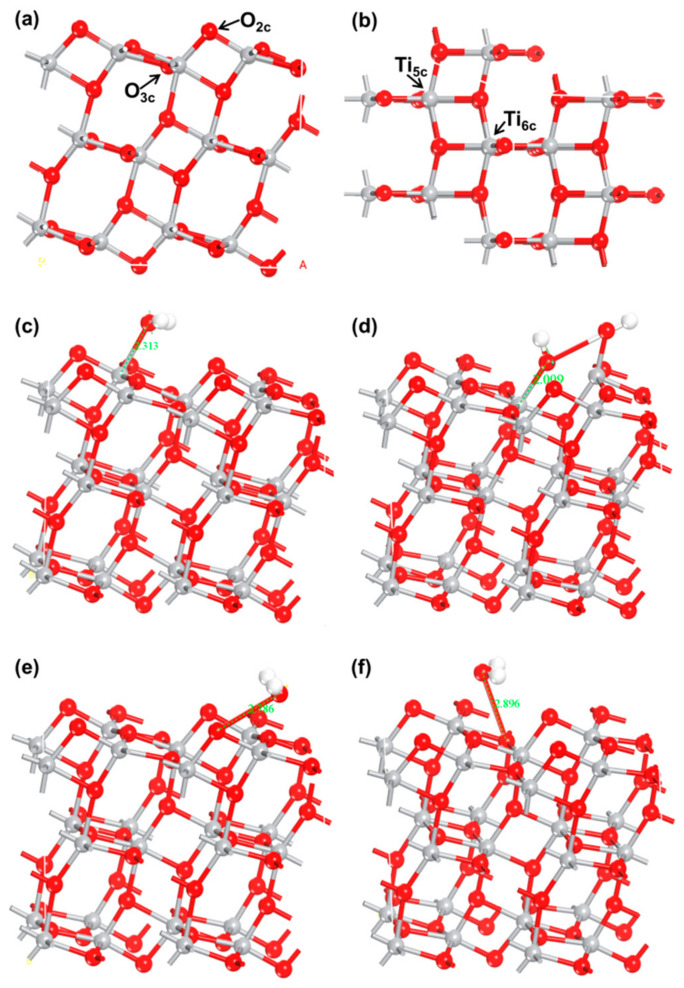
(**a**) Side view and (**b**) top view of optimized structures of anatase TiO_2_ (101) surfaces. Visualization of water-anatase TiO_2_ (101) configuration models with various adsorption sites: (**c**) Ti_5c_, (**d**) Ti_6c_, (**e**) O_2c_, and (**f**) O_3c_.

CNC is rich in hydroxyl groups, which makes H_2_O molecules easily adsorbed on the TiO_2_/CNC composite film, and the interwoven network and TiNP structure give more channels for H_2_O molecules. To elucidate the humidity sensing mechanism of TiO_2_/CNC-based humidity sensors, the interaction between H_2_O molecules and anatase TiO_2_ (101) surface was investigated. The optimized geometries of H_2_O molecules adsorbed on anatase TiO_2_ (101) surfaces with four adsorption sites are illustrated in Figure 7a,b.

The atomic structures were almost identical after H_2_O adsorption and surface relaxation, except those at the Ti_6c_ site. The adsorption structure diagram of Ti_6c_ revealed that one of the H atoms split from H_2_O and diffused to the adjacent bridging oxygen surface, indicating the formation of an O–H bond at the surface [33]. The adsorption energy (E_ads_), adsorption length (D_a_) (defined as the length between the gas molecules and the nearest atom on anatase TiO_2_ (101) surface), bond length (R_O-H1_ and R_O-H2_), bond angle (∠H–O–H), and band gap (E_g_) are summarized in Table 2.

The calculation results revealed that the adsorption energy of H_2_O molecules on the Ti_5c_ site of TiO_2_ surface was −0.693 eV, which is consistent with the experimental results (−0.72 eV) of previous studies [34]. The adsorption energies between H_2_O and anatase TiO_2_ (101) surfaces were characterized by negative E_ads_ values, which suggests that the exothermic process does not require additional energy, with the system becoming more stable after adsorption. Moreover, the adsorption energy value of H_2_O on the Ti_5c_ site was higher than those on other sites. The adsorption energy of the O_3c_ site was similar (−0.690 eV) to that of the Ti_5c_ site; however, the adsorption bond length of the O_3c_ site (2.896 Å) was significantly longer than that of the Ti_5c_ site (2.313 Å). Shorter bond lengths observed between H_2_O molecules and anatase TiO_2_ (101) surfaces imply that there were stronger interactions between H_2_O molecules and TiO_2_ surface, and therefore a more stable adsorption conformation. Furthermore, when H_2_O adsorption at the Ti_6c_ site causes a relatively large relaxation of the TiO_2_ structure, the Ti–O bond between the O atom below the TiO_2_ surface and the subsurface Ti atom is broken and the O atom position significantly shifts upward (Table 2). With regard to the Ti_6c_ site, the R_O-H2_ and ∠H–O–H values were significantly different when compared to other adsorption sites due to the breaking of the O–H bond. According to the results, Ti_5c_ was the most suitable site for H_2_O adsorption, and was used in subsequent calculations.

The calculated band gap values of TiO_2_ were lower than the actual value of 3.2 eV due to local and gradient density approximations based on DFT, which could underestimate band gap values of the system [35]. The band gaps of TiO_2_ were reduced to varying degrees after H_2_O adsorption when compared to the unadsorbed TiO_2_ (2.523 eV). The band gap of TiO_2_ decreased to 2.491 eV when H_2_O molecules were adsorbed on the Ti_5c_ site.

**Figure 8:**
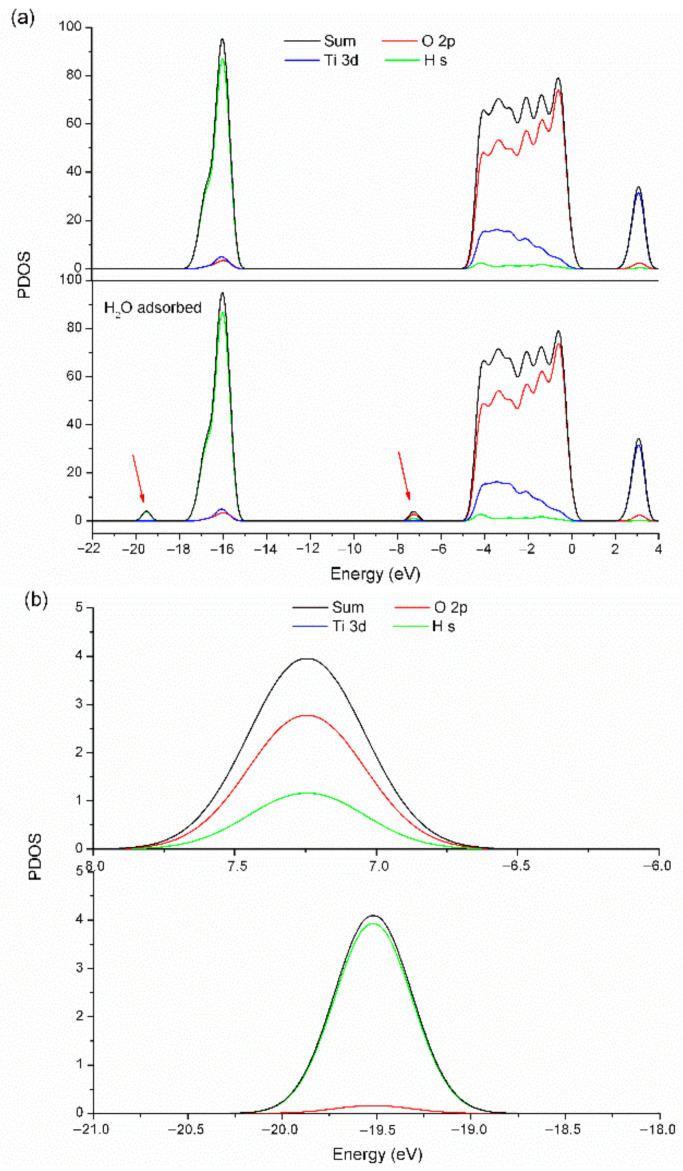
(**a**) Projected density of states of TiO_2_ atoms before and after adsorption of water molecules on anatase TiO_2_ (101) surfaces and (**b**) local enlargements of projected density of states of TiO_2_ atoms after adsorption of water molecules on anatase TiO_2_ (101) surfaces.

Therefore, response of the TiNP/CNC-based humidity sensor is stimulated by the chemical adsorption of H_2_O molecules on the Ti_5c_ site of anatase TiO_2_ (101) surface at the initial stage. To further reveal the effect of each subsystem on molecular orbital density, projected density of states (PDOS) of TiO_2_ surface after adsorption of H_2_O molecules are shown in Figure 8.

Figure 8a illustrates variations in density of states with H_2_O adsorption. New energy levels are formed in the energy band structure of TiO_2_ when H_2_O molecules are adsorbed. The chemisorption process of H_2_O molecules results in the formation of an adsorption complex (Figure 8b). The adsorption of H_2_O molecules causes migration of oxygen vacancy defects from the subsurface to the surface of TiO_2_ and induces dissociation of H_2_O molecules, in turn, resulting in the formation of surface OH groups (Equation (3)) [36,37].

2H_2_O→H_3_O^+^ + OH^-^ (3)

Subsequently, another H_2_O molecule is adsorbed through hydrogen bonding between two neighboring OH groups. The condensed topmost H_2_O molecules cannot move freely due to restrictions associated with the two hydrogen bonds [15]. During the process, only a few molecules were chemisorbed; therefore, the transfer of hydronium ions (H_3_O^+^) in the H_2_O layer becomes a challenge, which leads to low conductivity [38]. The TiNP/CNC-based humidity sensors exhibited low sensitivity under low humidity levels, which is consistent with the results illustrated in Figure 4.

During the following stage, H_2_O molecules are ionized under an electrostatic field, in turn, generating numerous H_3_O^+^ as charge carriers with an increase in humidity levels (Equation (4)) [39].

H_3_O^+^→H_2_O + H^+^ (4)

**Figure 9:**
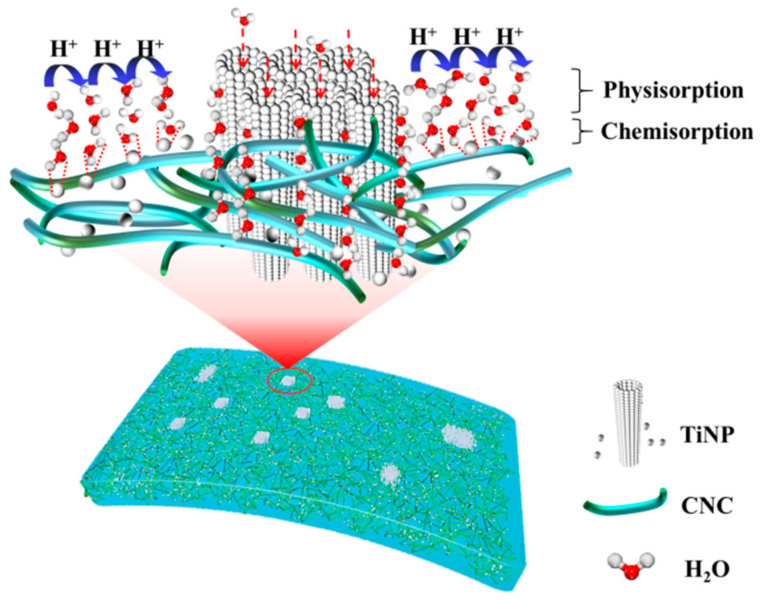
A schematic illustration of the humidity sensing mechanism of the TiNP/CNC-based humidity sensor.

Afterwards, increasing humidity levels lead to adsorption of more H_2_O molecules on the physisorbed layer, which behaves like a normal liquid. Therefore, the hopping of protons through adjacent H_3_O^+^ increases conductivity [40]. A further increase in humidity enhances the bonding of H_2_O molecules with OH groups to form a physisorbed layer, where carriers can be transferred easily, in turn, decreasing resistance [39]. In addition, the abundant surface oxygen vacancy defects can accelerate the decomposition capacity of H_2_O molecules, which accelerates response speed [9]. Therefore, when RH is greater than 50%, the response value of the sensors increases rapidly, as illustrated in Figure 4. A schematic presentation of the humidity sensing mechanism of TiNP/CNC-based humidity sensors is illustrated in Figure 9.

Notably, due to the presence of certain hydrophilic functional groups (OH^-^) on CNC surface, more H_2_O molecules can be adsorbed on the surface. After the multi-level nanostructured TiO_2_ is compounded with CNC, spherical TiO_2_ particles form a porous structure with adjacent CNCs, which has good adsorption capacity and facilitates the formation of a continuous H_2_O layer, in addition to serving as a direct conduction pathway for the improvement of the sensing membrane conductivity. Tubular TiO_2_ also provides several adsorption channels for H_2_O molecules, which is conducive to the rapid adsorption and desorption of H_2_O molecules. Therefore, TiNP/CNC composite nanostructures enhance humidity sensing properties.

## 4. Conclusions

Flexible humidity sensors composed of TiNP and CNCs were successfully prepared through electrochemical anodization and enzymatic hydrolysis combined with high pressure homogenization, followed by ultrasonic dispersion and vacuum evacuation. According to the results of FESEM, XRD, FTIR, and BET analyses, material characterizations of TiNP/CNC composite films were as follows. The average length and diameter of rod-shaped CNCs were 60 nm and 800 nm, respectively. TiNP consisted of two types of particles: 0-D spherical nanoparticles (average diameter of 60 nm) and 1-D residual nanotubes (average pore diameter of 200 nm) of TiO_2_. The crystal structure of TiNP was anatase phase, which was not altered after forming a composite with CNC. Furthermore, the surface area of TiNP/CNC composite films was 12.39 m^2^/g. The TiNP/CNC composite films had large specific surface area and superior hydrophilicity in combination with the results in FTIR spectra.

The humidity sensing characteristics of the TiNP/CNC-based humidity sensors with various TiNP contents (1–12 wt%) were analyzed. The results revealed that the 6% TiNP/CNC-based humidity sensor exhibited remarkable sensing characteristics, including high response in a wide RH detection range (11–97% RH), rapid response and recovery time, as well as high reproducibility and stability. This highlights the advantages of using TiNP/CNC composite films in the fabrication of high quality humidity sensors at room temperature. Furthermore, DFT calculations were performed to determine the sensing mechanism of TiNP/CNC composite films based on interaction with H_2_O molecules. The band gap, adsorption energy, and PDOS of optimized TiO_2_ were calculated before and after adsorption of H_2_O molecules.

The nanostructure of the TiNP/CNC composite films enhanced the humidity sensing properties of the sensors. The interlaced CNC and multidimensional TiNP structures have superior adsorption properties, which facilitate the formation of a continuous H_2_O layer and act as a direct conduction pathway for the enhancement of humidity sensing performance of the flexible TiNP/CNC composite films. In addition, TiNP/CNC-based humidity sensors confer the following advantages: flexibility for application in wearable devices, non-toxic for human use, low cost, and simple manufacturing technology, in addition to degradability and non-polluting properties. Therefore, flexible humidity sensors fabricated from TiNP/CNC composite films could be a potential strategy for developing multifunctional humidity sensors due to their high response and wide RH detection range capacities.

## Figures and Tables

**Table 1:** Comparison of different flexible humidity sensor based on TiO_2_.

Material	Humidity Range (% RH)	Response (R_0_/R)	Response/Recovery Time	Substrate	References
TiO_2_	0–70	10	3 min/50 s	PET *	[29]
TiO_2_ nanowire network	30–75	-	3.6 s/14 s	PET *	[30]
TiO_2_ nanoflower	20–95	10^3^~10^4^	hundreds of second	PI *	[31]
TiO_2_ nanowire	20–90	-	4.5 s/2.8 s	ITO *	[32]
TiO_2_ nanoparticles	11–97	9.7-1900	34 s/18 s	CNC	This work

* PET: poly-ethylene terephthalate, PI: polyimide, ITO: indium tin oxide.

**Table 2:** Geometrical and electronic parameters of H_2_O-adsorbed models with various adsorption sites: adsorption energy (E_ads_), adsorption length (D_a_), defined as the length between the gas molecules and the nearest atom on anatase TiO_2_ (101) surface, bond length (R_O-H1_ and R_O-H2_), bond angle (∠H–O–H), and band gap.

Adsorption Site	E_ads_(eV)	D_a_(Å)	R_O-H1_ (Å)	R_O-H2_ (Å)	∠H-O-H (°)	Band Gap (eV)
Ti_5c_	−0.693	2.313	0.984	0.980	105.103	2.491
Ti_6c_	−0.403	2.009	0.976	3.594	78.150	2.507
O_2c_	−0.603	2.786	0.979	0.979	106.205	2.511
O_3c_	−0.690	2.896	0.983	0.981	104.537	2.484
